# Parental emotional expression and children's oral narrative ability: a systematic review

**DOI:** 10.3389/fpsyg.2025.1629744

**Published:** 2026-01-12

**Authors:** Zhi Hong Zhang, Suziyani Mohamed, Kamariah Abu Bakar

**Affiliations:** Faculty of Education, Universiti Kebangsaan Malaysia, Selangor, Malaysia

**Keywords:** parents, emotional expression, children's oral narrative, bibliometric analysis, systematic review

## Abstract

This study aims to systematically examine the relationship between parental emotional expression and children**'**s oral narrative abilities, as well as the developmental trends in this research field. Based on the Scopus database and adhering to PRISMA principles, 81 articles published between 2010 and 2025 were selected. A combined approach of bibliometric analysis and thematic analysis was employed for the investigation. The bibliometric analysis revealed an overall upward trend in research activity within this field, with peaks in 2015 and 2021. Recent years have witnessed shifts in research focus and methodologies. Themes have gradually expanded from general language development to emotional socialization and family interactions, demonstrating interdisciplinary convergence. Thematic analysis indicates that parental emotional expression influences children's oral narrative development through multiple pathways, including the family literacy environment, digital devices, parent-child interaction, and cultural background. Positive emotional expression and high-quality parent-child interactions promote language richness and emotional depth in children's narratives, whereas negative or absent emotional expression may inhibit their language organization abilities. Research indicates that parental emotional expression exerts both direct and indirect effects on children's narrative abilities. Future studies should strengthen interdisciplinary collaboration by integrating perspectives from psychology, education, and digital technology to deepen understanding of the mechanisms through which the family emotional environment influences children's language development.

## 1 Introduction

Preschool is a critical period for the development of children's oral narrative ability. Oral narrative ability is the ability to express language in an organized manner out of context (Bailey et al., 2020). Miles and Chapman (2002) define oral narrative ability as the ability to understand, interpret, and retell stories accurately and meaningfully. Proficient language and communication skills are fundamental for individuals to adapt successfully to society. Narrative competence, as a crucial aspect of children's language development, significantly influences their social, cognitive, and psychological development (Gardner-Neblett, 2022; Hidayat and Fitria, 2021; van der Wilt et al., 2022). In recent years, more and more researchers have begun to pay attention to the development of children's oral narrative abilities. For example, Scott et al. (2022) and Johnson et al. (2023) successively designed a new systemic framework for evaluating children's oral narrative abilities. By extracting features from recordings of children retelling stories, this framework enables fair assessments of children's language skills to support and enhance teaching practices. Additionally, Lucero (2018) focused on analyzing the impact of bilingual education on children's oral narrative abilities. Research findings indicate a significant correlation between children's oral narrative performance across languages, with bilingual children demonstrating distinct advantages over monolingual peers. However, Clark-Whitney and Melzi (2023) shifted focus to examining the influence of executive function on oral narrative abilities in bilingual children. Their study revealed that executive function more effectively promotes the development of both oral narrative skills and early reading abilities in children.

Oral narrative ability typically encompasses two categories: personal narratives and fictional narratives (Allen et al., 1994; Kim, 2018). The former is based on children's autobiographical memories, primarily involving the organization and expression of real-life events; the latter relies on imaginative construction, emphasizing creativity and plot structure. However, this study focuses on children's personal narrative ability, as personal narratives more directly reflect children's socialization experiences and emotional processing within the family's emotional environment. Parents' emotional expressions typically occur within authentic interactive contexts and exert a critical influence on how children narrate their own experiences.

The family is an important environment for children's growth and emotional enlightenment, providing emotional support and security, and is an initiating place for children's language development, even though a systematic school curriculum cannot be separated from the support and cooperation of family emotional education (Buschmann, 2020; Göksun et al., 2025; Roostin, 2018). It has been shown that a family's socioeconomic status, the availability of learning resources, parenting styles, and level of education affect the development of children's language communication abilities (Fang et al., 2018; Qi and Wu, 2020; Schwab and Lew-Williams, 2016). Family support for children's language and learning and the resources provided are beneficial in promoting children's language communication abilities (Cohen et al., 2020; Zheng et al., 2021). Family support and resources refer to parental involvement in children's learning, access to educational materials at home, and emotional encouragement provided by caregivers (Fuller et al., 2019; Otero-Mayer et al., 2025). These factors are considered key environmental influences on children's language and narrative development.

Parental emotional expression refers to the primary mode or style of verbal and non-verbal expression exhibited by parents in the home, i.e., how emotions are expressed and conveyed through words, facial expressions, body language, etc., (Tang et al., 2023). The core function of emotional expression is to communicate needs and wants, initially realized within the family and then extended to the wider social environment, where its form, intensity, and inhibition change depending on the context (Keltner et al., 2019). Parents are crucial to the development of children's oral narrative abilities, in which emotional expression can intuitively serve as a stimulus to the development of children's oral narrative abilities (Leyva et al., 2014; Masters et al., 2022). Parental emotional expression influences language development through the family literacy environment, thus affecting children's narrative ability. Researchers usually consider the family literacy environment as factors in the home that influence literacy development and define it in different dimensions (Dong et al., 2020; Torppa et al., 2022).

Romero-González et al. (2023) argues that the family literacy environment affects children's reading ability through interpersonal interactions, the physical environment of the home, and motivational atmosphere. Fish and Pinkerman (2003) summarized the family literacy environment in terms of three dimensions: contextual variables, child characteristics, and parent-child interactions. Some scholars have proposed specific components for the home reading and writing environment. Pietropoli and Gracia (2025) argue that it should encompass the quality of family activities, material resources, and the social environment. Fekonja-Pekljaj et al. (2010) share a similar perspective, suggesting that the family literacy environment should include the quality of family activities, physical resources, and the social context. In addition, Sénéchal and LeFevre (2002), as well as Puglisi et al. (2017) categorized the family literacy environment into two formal literacy activities and informal literacy activities dimensions, where formal literacy activities are those in which adults directly teach reading or improve print-related skills at home, and informal literacy activities are those in which children engage in reading or are directed to pay attention to print in the environment. As digital technology has long been immersed in family life, it has led to an increase in children's use of smartphones and tablets (Holloway et al., 2013). As a result, family literacy environments are no longer limited to traditional print activities. Based on the above points, this study defines the family literacy environment as a multidimensional concept encompassing interpersonal interactions within the family, the material and physical resources of the family environment, the sociocultural context, formal and informal literacy activities, and the influence of digital technology integration into family life.

Previous review studies have primarily focused on multifaceted aspects within the domain of factors influencing child development. Research by Liu and Zhao (2024) underscored the significance of these factors in child development, yet did not specifically examine the impact of parental emotional expression on children's oral narrative abilities. Empirical research on the effects of parental emotional expression on children's oral narrative abilities remains extremely limited, with only a handful of studies such as Sher-Censor and Yates (2015) venturing into this specific domain. In Western countries, particularly the United States, Canada, and some European nations, a substantial body of empirical research exists on the effects of family environments on children's language and cognitive development. In contrast, empirical research in China has primarily focused on the effects of parenting styles on children's language development (Yanxue and Bhaumik, 2024; Yu et al., 2024), the effects of family socioeconomic status on children's language and cognitive development (González et al., 2024; Madya et al., 2019), and the impact of bilingual environments on language development (Zhang et al., 2023).

**Figure 1:**
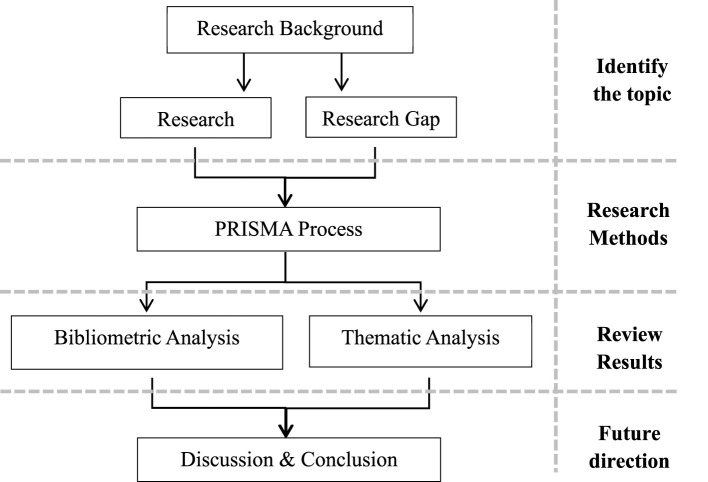
The operational framework of this study.

Although an increasing number of studies have examined the relationship between parental emotional expression and children's oral narrative ability, existing empirical research is mostly based on Western studies, neglecting the cultural heterogeneity of Asian societies in parenting practices and child development; In addition, the research results on the relationship between parental emotional expression and children's oral narrative ability are still remain fragmented, and there is no comprehensive review has systematically synthesized this relationship. Based on this, the purpose of this research systematic review is (i) to systematically organize and synthesize existing literature on the relationship between parental emotional expression and children's oral narrative abilities in the Asian countries, thereby clarifying the current state of research and its primary conclusions of Asian culture; (ii) to identify research hotspots and developmental trends in this field in the Asian countries through bibliometric analysis; and (iii) to summarize theoretical and methodological shortcomings in existing studies in the Asian countries, thereby providing direction for future regional targeted empirical research. The operational framework of this study is shown below in Figure 1.

## 2 Data and methods

This article follows the PRISMA guidelines for conducting and reporting literature reviews. It mainly includes identification the articles, screening the articles, and eligibility and included the articles.

### 2.1 Identification

This study utilized the Scopus database, which is recognized as one of the most authoritative and comprehensive academic databases globally. It encompasses over 294 research areas (Yoon et al., 2023). According to the Scopus database, a total of 26 journals related to parental emotional expression and children's oral narrative ability are indexed in it, indicating its strong representation and academic depth.

Although existing studies have suggested combining multiple databases to expand the coverage of literature (Younger, 2010), in this study, Scopus was finally selected as the only core data source due to the considerations of research focus, data processing efficiency, and resource integration. To ensure the systematic and comprehensive nature of the search, the search terms used were: TITLE-ABS-KEY (((“parents” OR “caregivers” OR “guardians”) AND (“emotional expression” OR “affect” OR “emotion” OR “feelings”) AND (“children” OR “youth” OR “kids” OR “offspring”) AND (“oral narrative” OR “storytelling” OR “narrative ability” OR “verbal skills”) AND (“development” OR “skills” OR “competence” OR “literacy”))) AND PUBYEAR > 2009 AND PUBYEAR <2026 AND (LIMIT-TO (LANGUAGE, “English”)) AND (LIMIT-TO (DOCTYPE, “ar”) OR LIMIT-TO (DOCTYPE, “cp”) OR LIMIT-TO (DOCTYPE, “re”)). A total of 81 potentially relevant documents were retrieved in Scopus.

In addition, in order to try to compensate for the possible coverage limitations of a single database, this study synchronously referred to important research findings mentioned in existing reviews and highly cited literature in the field, to provide more in-depth insights into the research on parental emotional expression and children's oral narrative ability. After this supplementary search, no further relevant articles were identified beyond those already retrieved through the initial database search. Therefore, a total of 81 articles were selected. These selected articles will be utilized in bibliometric analysis, providing comprehensive and reliable data support for in-depth examination of the field's document profiles, publication trends, publications by authors, publications by countries, and top keywords. See Table 1 for details.

**Table 1:** Keywords used for search and number of papers retrieved.

**1st step**	
**Item**	**Details**
Keywords	“parents,” “caregivers,” “guardians”
	“emotional expression,” “affect,” “emotion,” “feelings”
	“children,” “youth,” “kids,” “offspring”
	“oral narrative,” “storytelling,” “narrative ability,” “verbal skills”
	“development,” “skills,” “competence,” “literacy”
Operators	“OR,” “AND”
Period	2010-2025
Language	English
Data source	Scopus
Search date	March 2025
Total number	81

### 2.2 Screening

In order to maintain the high quality of the content and to ensure a manageable number of articles were selected, the search for articles was limited to “papers,” “published articles” and “refereed articles,” “conferences,” and “scholarly papers” published in peer-reviewed journals, as this is the main source of empirical data. Only English-language resources were included in the review. All articles published between 2010 and 2025. Most importantly, articles published in the field of “Developmental and Educational Psychology” and “Education” were selected in order to increase the likelihood of retrieving relevant articles. In addition to this, after careful consideration of existing literature and research objectives, we found that studies focused on the Asian region can explore research objectives in a more in-depth and targeted manner, offering valuable insights that research in other regions may not fully capture. Therefore, all studies from Asian countries were included in the screening of the article, while articles from non-Asian countries were excluded. A total of 14 articles were retained at this stage (see Table 2).

**Table 2:** The inclusion and exclusion criteria.

**Criteria**	**Inclusion**	**Exclusion**
Article type	Research article, review article, conference paper	Book, book chapter, erratum, editorial
Language	English	Non-English
Year	2010-2025 year	Less than 2010 year
Country/region	Asian countries	Non-Asian countries
Subject areas	Developmental and educational psychology, education	Non-developmental and educational psychology, education

### 2.3 Eligibility and included

**Figure 2:**
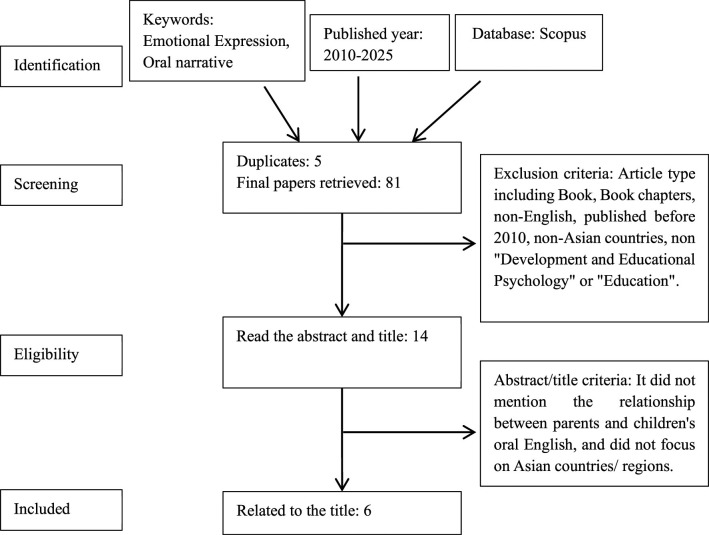
PRISMA flow diagram for the systematic review (Moher et al., 2009).

The third stage is eligibility stage. Based on the articles retrieved from the database in Method 2.2, all abstracts and the main content of the articles of the 14 original samples were read to ensure that they met the inclusion criteria. Any articles relating to the relationship between parents and children's oral language were retained. The tendency was to retain articles that contained descriptive ambiguity in their abstracts because the literature on parental emotions and children's oral narrative ability is extensive and covers many different dimensions, such as parental emotional expression, parental emotional transmission, parental emotional response, family interactions, children's creativity, children's emotional development, and so on. Therefore, no inclusion/exclusion criteria were used other than whether the papers included both parental emotion and children's narrative ability topics in their abstracts. Similarly, articles that did not focus on Asian countries and regions were similarly excluded. After screening, the number of relevant articles has been reduced to six. Please refer to Table 3 for details. These selected studies will serve as the foundation for thematic analysis, providing rich data and case support for subsequent theme extraction. The methodological framework for the review is shown in Figure 2.

**Table 3:** List of studies included in the systematic review.

**No**.	**Authors**	**Published year**	**Title**	**Study design**	**Country of focus**	**Key findings**	**Database type**
1	Debarati M. et al.	2024	Digital tools for direct assessment of autism risk during early childhood: a systematic review	Review	Israel, United States, Australia, etc.	Evaluating the risk of autism in children using digital tools can be achieved by recording and analyzing their interactions and verbal responses with digital interfaces. Indirectly reflecting the role of family interaction patterns in children's oral development	Scopus
2	Anita W. et al.	2011	Development of the language subtest in a developmental assessment scale to identify Chinese preschool children with special needs	Questionnaire	Hong Kong, China	Develop and validate the language subtest of the Preschool Developmental Assessment Scale (PDAS), and compare and analyze the differences in language abilities between children with Special Educational Needs (SEN) and ordinary children through questionnaire research on children	Scopus
3	Sebastian et al.	2019	Parents, Television, and Children's Emotional Expressions: A Cross-Cultural Multilevel Model	Questionnaire, interview	China, Malaysia, Thailand, etc.	Using a cross-cultural multilevel model, we analyzed self-reported data from 3,570 children aged 6–15 from 13 different countries (Asia, Europe, Middle East, and South America) to explore the impact of parents and television on children's emotional expression	Scopus
4	Hong et al.	2021	Meta-Analysis of RCTs of Technology-Assisted Parent-Mediated Interventions for Children with ASD	Review	Israel, United States, Australia, etc.	Technology assisted parent mediated interventions may be effective in improving emotional recognition in children with autism, but no significant differences were observed in social communication, social functioning, and language outcomes. Currently, there is no single technology assisted parent mediated intervention that fully meets evidence-based intervention standards	Scopus
5	Wen et al.	2024	Association between home literacy environment and eye movements during storybook reading: implications for vocabulary and word reading in Chinese preschoolers	Questionnaire	China	Interactive reading is the only significant component of the family reading and writing environment that affects children's attention to text and word reading. Children's attention to text is significantly correlated with their vocabulary and word reading ability, indicating that more attention to text can help improve reading ability. Although other components of the family's reading and writing environment (such as language stimulation, reading books, collaborative activities and conversations, and zone of proximal development stimulation) are associated with certain aspects of reading ability, interactive reading is most prominent in promoting children's attention to text and word reading	Scopus
6	Hsieh, M.-F.	2025	This is My Favorite Book': Exploring Children's Perspectives on Books and Reading	Interview	Taiwan, China	Children have a wide range of preferences for book types, including storybooks, information books, and animated adaptations. They actively participate in reading, understand and share their interests, and express their opinions. Children cherish the time spent reading with adults, believing that it can enhance reading comprehension and bring joy. At the same time, they hope to have more opportunities to choose books and participate in purchasing decisions. These findings emphasize the initiative of children in reading and the need for adult support	Scopus

Given the limited number of studies ultimately included, this research employed a narrative thematic synthesis approach rather than inductive thematic analysis involving line-by-line coding. The researchers conducted multiple rounds of repeated readings of all included studies to familiarize themselves with their research focus, core conclusions, and conceptual frameworks. Through repeated comparisons across the literature, they identified recurring viewpoints and patterns. Subsequently, these shared elements were integrated and elevated to form higher-level descriptive themes.

## 3 Review results

The analysis focused on two core areas: an in-depth bibliometric analysis of 81 documents indexed in the Scopus database, and a systematic thematic analysis of these documents on the topic of parental emotional expression and children's oral narrative ability. Firstly, at the level of bibliometric analysis, 81 articles were selected from Section 2.1 as the dataset to analyze their literature, publication trends, co authors, and countries. Our aim is to reveal the research hotspots and future research trends in this field. Secondly, in terms of thematic analysis, the six articles selected in Section 2.3 were treated as a dataset to explore the intrinsic relationship between parental emotional expression and children's oral narrative ability in published articles. Through careful combing and summarization of the literature content, it was classified as the influence of home literacy environment, digital devices, parent-child interaction, and cultural background factors on children's oral narrative ability.

### 3.1 Bibliometric analysis

This study conducted a bibliometric analysis based on 81 articles retrieved from the Scopus database, primarily utilizing Excel and VOS viewer software. Following the Scopus search, a Worldwide Scientific Production Map was automatically generated to analyze publication distribution by country. When employing VOS viewer, the analysis type was set to co-occurrence analysis of author keywords to identify research hotspots and keyword associations, with a threshold of 3 applied to ensure keyword representativeness. Other analyses—including publication distribution by author, publication trends, and literature overview—were processed using Excel.

#### 3.1.1 Documents profiles

Among the documents screened under the theme of this study, Article, as the most dominant form of publication in the field, accounted for 83.95% of the total number of publications, which was significantly higher than Conference Paper (12.35%) and Review (3.70%; see Table 4). This result suggests that Article, as the core carrier of original research results, dominates in scholarly communication and knowledge accumulation. Articles usually adopt a strict peer-review mechanism and follow a systematic research methodology and theoretical framework (Lim, 2021), so their high percentage reflects the systematic and scientific nature of research in the field. In contrast, Conference Paper is mainly used for communication of cutting-edge research in academic conferences (Lee, 2019), and its relatively small number may indicate the relatively limited conference communication of this research topic. Meanwhile, Reviews accounted for only 3.70%, indicating that the systematic organization and integration of existing research results in this field is limited.

**Table 4:** Document type.

**Document type**	**Total publications (TP)**	**Percentage (%)**
Article	68	83.95
Conference paper	10	12.35
Review	3	3.70

Table 5 reveals the distribution of different publication sources. The study shows that Journal is the most important source of publication in this field, accounting for 86.42%, far exceeding Conference Proceeding (11.11%) and Book Series (2.47%). This trend further validates the centrality of journals in the dissemination and accumulation of scholarly results. Articles have become the main publication channel for researchers in the field due to their long review cycle, high academic rigor and wide academic influence. In contrast, Conference Proceedings still play an important role in promoting academic communication and presenting the latest research results, but their influence is usually not as high as that of high-quality journals, and Book Series accounts for the lowest proportion of 2.47%, which indicates that the results of this research topic are rarely published in the form of scholarly monographs or book series. However, as an important carrier of systematic reviews, theoretical innovations and interdisciplinary research, book series still have a certain academic value for the accumulation and dissemination of knowledge in this field.

**Table 5:** Source type.

**Source type**	**Total publications (TP)**	**Percentage (%)**
Journal	70	86.42
Conference proceeding	9	11.11
Book series	2	2.47

Table 6 shows the distribution of publications in different subject areas, revealing the research hotspots and disciplinary diversity of the topic of parental emotional expression and children's oral narrative abilities. Medicine (39.51%) and psychology (37.04%) together constitute the core areas of this research topic with a relatively high percentage. This indicates that the research on this topic focuses mainly on the medical and psychological perspectives of children's language development and parents' emotional expression. In addition, social sciences (18.52%) served as the third most important area of study, reflecting the importance of this theme in educational psychology, family relationships, and social development. In addition to the core disciplines, the higher share of computer science (13.58%) indicates that more and more studies try to utilize technological tools such as Artificial Intelligence and Natural Language Processing to analyze children's narrative abilities or the characteristics of parents' emotional expressions. For example, computer-assisted analysis tools may be used to automatically assess children's oral expression (Hu et al., 2022; Sinclair et al., 2021) or to identify and quantify patterns of parental emotional expression (Narimisaei et al., 2024; Tuyen et al., 2017). Meanwhile, nursing (8.64%), health sciences (7.41%), and neuroscience (7.41%) also suggest that the topic is expanding into the broader field of health and cognitive sciences. This may involve early intervention (Custode et al., 2024; Hou, 2022; van der Wilt et al., 2019), child mental health (Conway et al., 2021; Osborne and Ahinkorah, 2024; Wang, 2023), and how neural mechanisms (Benítez-Barrera et al., 2024; Nguyen, 2023; Romeo et al., 2018; Tulling, 2022; Zhai et al., 2023) affect children's language abilities. A small number of studies involved biochemistry, genetics and molecular biology (4.94%) exploring the influence of genetic factors. Arts and humanities (3.70%), on the other hand, focused on the cultural dimension of narrative expression. The multidisciplinary nature of the studies not only contributes unique perspectives to the field but also increases the diversity of research.

**Table 6:** Subject area.

**Subject area**	**Total publications (TP)**	**Percentage (%)**
Medicine	32	39.51
Psychology	30	37.04
Social sciences	15	18.52
Computer science	11	13.58
Nursing	7	8.64
Health professions	6	7.41
Neuroscience	6	7.41
Biochemistry, genetics and molecular biology	4	4.94
Arts and humanities	3	3.70
Environmental science	3	3.70
Mathematics	3	3.70
Decision sciences	2	2.47
Engineering	2	2.47
Pharmacology, toxicology and pharmaceutics	2	2.47
Physics and astronomy	2	2.47
Dentistry	1	1.23
Energy	1	1.23
Materials science	1	1.23

#### 3.1.2 Publication trends

**Figure 3:**
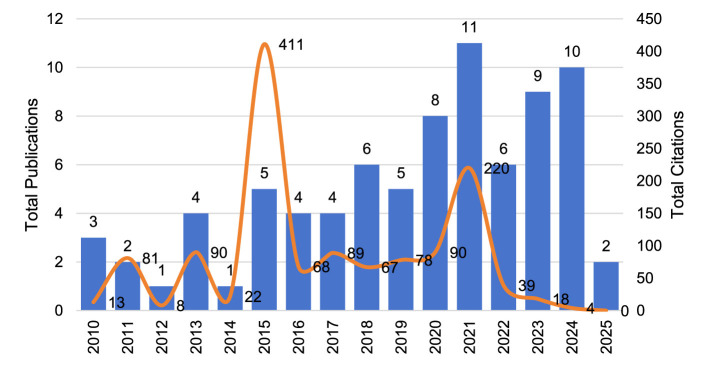
Total publications and citations by year.

From the data in Figure 3 and Table 7, the number of publications on this topic shows a fluctuating growth trend in general. 2010–2014 has a low publication volume, with an average of 2.75 articles, which is the initial stage. 2015 has become a turning point for the increase of the topic's attention. 2015–2019 has an average of 4.8 articles, which is a smooth development stage. Among them, the TC (total citations) in 2015 reached 411, and the C/P (average citations per publication) was as high as 82.20, indicating that the studies published in that year had high academic impact. 2020 present, the average number of articles is 7.67, which is a high growth stage. The TP (11) and TC (220) in 2021 show that some of the research results in that year had far-reaching impacts in the academic community, thus enhancing the overall impact of the topic. After 2022, although the publication volume is still high, its academic impact declines significantly, with the TC of only 18 in 2023 and the C/P dropping to 2.00, indicating that the citation rate of the research in this field and its academic impact are declining. 2022–2024, the average number of publications is 8.33, which is a high growth phase. The data for 2024–2025 suggests that the field may be in a phase of adjustment, especially as 2025 is only incomplete data as of March, and future trends still need to be further observed, and in the future the topic may further consolidate its position in the academic community as more research is conducted and new publications appear.

**Table 7:** Year of publication.

**Year**	**TP**	**NCP**	**TC**	**C/P**	**C/CP**	***h***	***g***
2010	3	2	13	4.33	6.50	2	3
2011	2	2	81	40.50	40.50	2	2
2012	1	1	8	8.00	8.00	1	1
2013	4	4	90	22.50	22.50	3	4
2014	1	1	22	22.00	22.00	1	1
2015	5	5	411	82.20	82.20	4	5
2016	4	3	68	17.00	22.67	3	4
2017	4	3	89	22.25	29.67	3	4
2018	6	5	67	11.17	13.40	3	6
2019	5	5	78	15.60	15.60	5	5
2020	8	7	90	11.25	12.86	5	8
2021	11	10	220	20.00	22.00	7	11
2022	6	6	39	6.50	6.50	3	6
2023	9	6	18	2.00	3.00	3	3
2024	10	3	4	0.40	1.33	1	1
2025	2	0	0	0.00	0.00	0	0
Total	81	63	1,298	16.02	20.6		

TP, total number of publications; NCP, number of cited publications; TC, total citations; C/P, average citations per publication; C/CP, average citations per cited publication; h, h-index; g, g-index.

#### 3.1.3 Publications by authors

From the data in Tables 8, 9 the academic output of this research field has a certain degree of authorship concentration, and at the same time shows the trend of multi-authorship cooperation. These authors come from different countries and regions, showing that this research field is characterized by internationalization. Among the prolific authors, Pace, C.S. (Italy), Muzi, S. (Italy), and Tenenbaum, H.R. (UK) are the most influential researchers with two publications in the field and high citation counts (TC all more than 50), respectively. In addition, a number of authors are from the UK, USA, Italy, India, Iran, Japan and other countries, showing that the research in this field has some internationalization characteristics, but is still dominated by scholars from Europe and the USA. Some authors (e.g., Aier, A. and Akard, T.F.) have higher average citation rates (C/P), indicating that their research may have greater academic influence.

**Table 8:** Top 25 most productive authors.

**Author's name**	**Affiliation**	**Country**	**TP**	**NCP**	**TC**	**C/P**	**C/CP**	***h***	***g***
Pace, C.S.	University of Genoa	Italy	3	3	51	17	17	3	0
Aznar, A.	Kingston University	United Kingdom	2	2	69	34.5	34.5	2	0
Muzi, S.	University of Genoa	Italy	2	2	14	7	7	2	0
Tenenbaum, H.R.	University of Surrey	United Kingdom	2	2	69	34.5	34.5	2	0
Abeyweera, R.	University of Colombo	Sri Lanka	1	1	5	5	5	1	0
Abid, M.	Duke University	United States	1	0	0	0	0	0	1
Acierno, S.P.	Seattle Children's Hospital	United States	1	1	8	8	8	1	0
Aier, A.	St. John's Medical College	India	1	1	13	13	13	1	0
Akard, T.F.	Vanderbilt University	United States	1	1	4	4	4	1	0
Al-Ammouri, I.	The University of Jordan	Jordan	1	0	0	0	0	0	1
Ambad, R.	Shalinitai Meghe Institute of Medical Sciences	India	1	0	0	0	0	0	1
Aminabadi, N.A.	Tabriz University of Medical Sciences	Iran	1	1	20	20	20	1	0
An, P.	Southern University of Science and Technology	China	1	0	0	0	0	0	1
Anagnostou, E.	University of Toronto	Canada	1	1	41	41	41	1	0
Ananthanarayan, S.	University of Oldenburg, Oldenburg	Germany	1	1	34	34	34	1	0
Anderson, E.	StoryAidEU	United Kingdom	1	1	3	3	3	1	0
Angelini, J.	University of Modena and Reggio Emilia	Italy	1	1	1	1	1	1	1
Annesi-Maesano, I.	/	France	1	1	35	35	35	1	0
Anyango, H.O.	African Population and Health Research Center (APHRC)	Kenya	1	1	12	12	12	1	0
Anziom, B.	L'Association pour la Traduction, l'Alphabétisation, et le Développement Holistique de l'Etre Humain (ASTRADHE)	Cameroon	1	1	8	8	8	1	0
Asano, K.	Tohoku University	Japan	1	1	31	31	31	1	0
Asano, M.	Tohoku University	Japan	1	1	31	31	31	1	0
Asiri, M.S.	Sulu State College	Philippines	1	0	0	0	0	0	1
Asta, L.	University of Modena and Reggio Emilia	Italy	1	1	1	1	1	1	1
Avello, E.	Pontificia Universidad Católica de Chile	Chile	1	1	13	13	13	1	0

TP, total number of publications; NCP, number of cited publications; TC, total citations; C/P, average citations per publication; C/CP, average citations per cited publication; h, h-index; g, g-index.

**Table 9:** Frequency of author collaboration.

**Author count**	**Frequency**	**Percentage (%)**
1	7	8.64
2	10	12.35
3	12	14.81
4	14	17.28
5	9	11.11
6	13	16.05
7	6	7.41
8	4	4.94
9	3	3.70
10	1	1.23
14	1	1.23
31	1	1.23
Total	81	100

No author is listed.

Table 9 reflects the co-authorship of papers in this field, with relatively few single-authored papers (8.64%) and the highest percentage of co-authored papers with 3–5 authors (14.81%−17.28%), suggesting that this research topic favors the collaboration of small research teams. The percentage of papers with more than 10 authors is extremely low (1.23%−1.23%), indicating that large-scale collaborative research is less likely to occur in this area. As the number of authors increases, the number of documents increases and then decreases, showing an inverted U-shaped distribution trend. These data reflect that moderate-sized teams (e.g., 3–6 people) in academic collaborations may be more conducive to efficiently producing research results. The high number of citations not only represents the influence of the authors' research results in the academic community, but may also mean that these studies have made important contributions to the development of the field.

#### 3.1.4 Publications by countries

**Figure 4:**
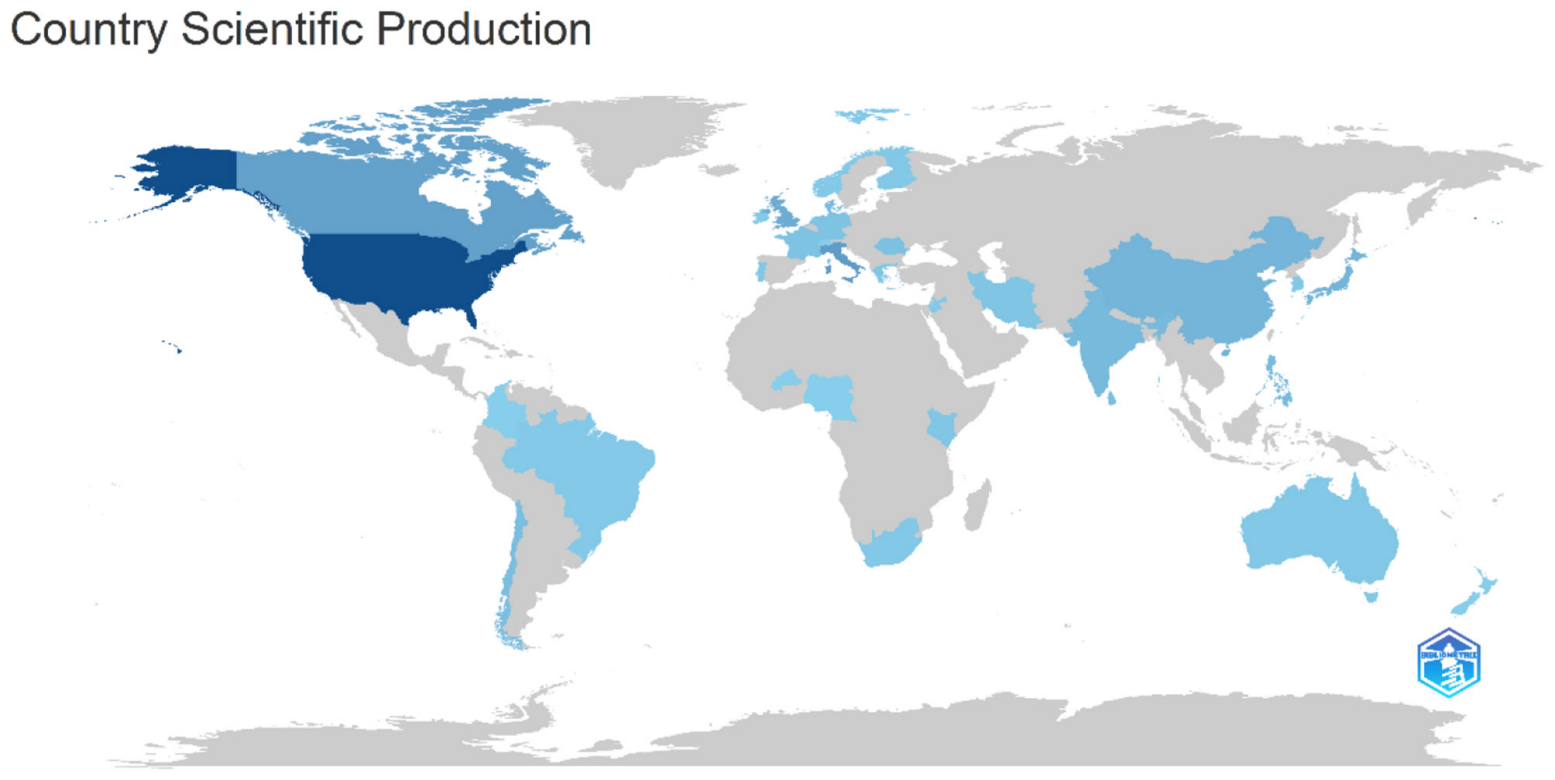
Worldwide scientific production map indexed by Scopus.

From the data in Table 10 and Figure 4 of the World Research Output Map, the academic contributions in this research field are mainly concentrated in the United States (88 articles), Italy (33 articles), and Canada (30 articles), which shows that North American and European countries dominate the research in this field. The United Kingdom (22 articles) and China (17 articles) also have high academic outputs, indicating that the topic has received more attention in English-speaking countries and some Asian countries. The relatively high research outputs from Asian countries such as Japan (16 articles), India (14 articles), and the Philippines (13 articles) indicate that the theme has a certain degree of academic impact on a global scale. In addition, emerging countries such as Chile (11 articles), and Sri Lanka (10 articles) have also contributed to this research, reflecting the gradual participation of scholars from developing countries in this field. The research map further validates this distribution trend, with the United States having the most significant research output globally, and growing research contributions from Europe, Asia, and some Latin American and African countries. Overall, the research field is dominated by Europe and the United States, while showing a trend of multinational participation and global cooperation, which may further expand to developing countries in the future. Gray-marked countries have relatively low or zero output in terms of scientific publications, and they need more support and attention to promote their scientific research endeavors.

**Table 10:** Top 20 Countries contributed to the publications.

**Country**	**Freq**
USA	88
Italy	33
Canada	30
UK	22
China	17
Japan	16
India	14
Denmark	13
Philippines	13
Chile	11
Sri Lanka	10
Romania	9
France	8
Germany	8
Iran	7
Netherlands	7
Greece	6
Kenya	6
Australia	5
Finland	5

#### 3.1.5 Top keywords

**Figure 5:**
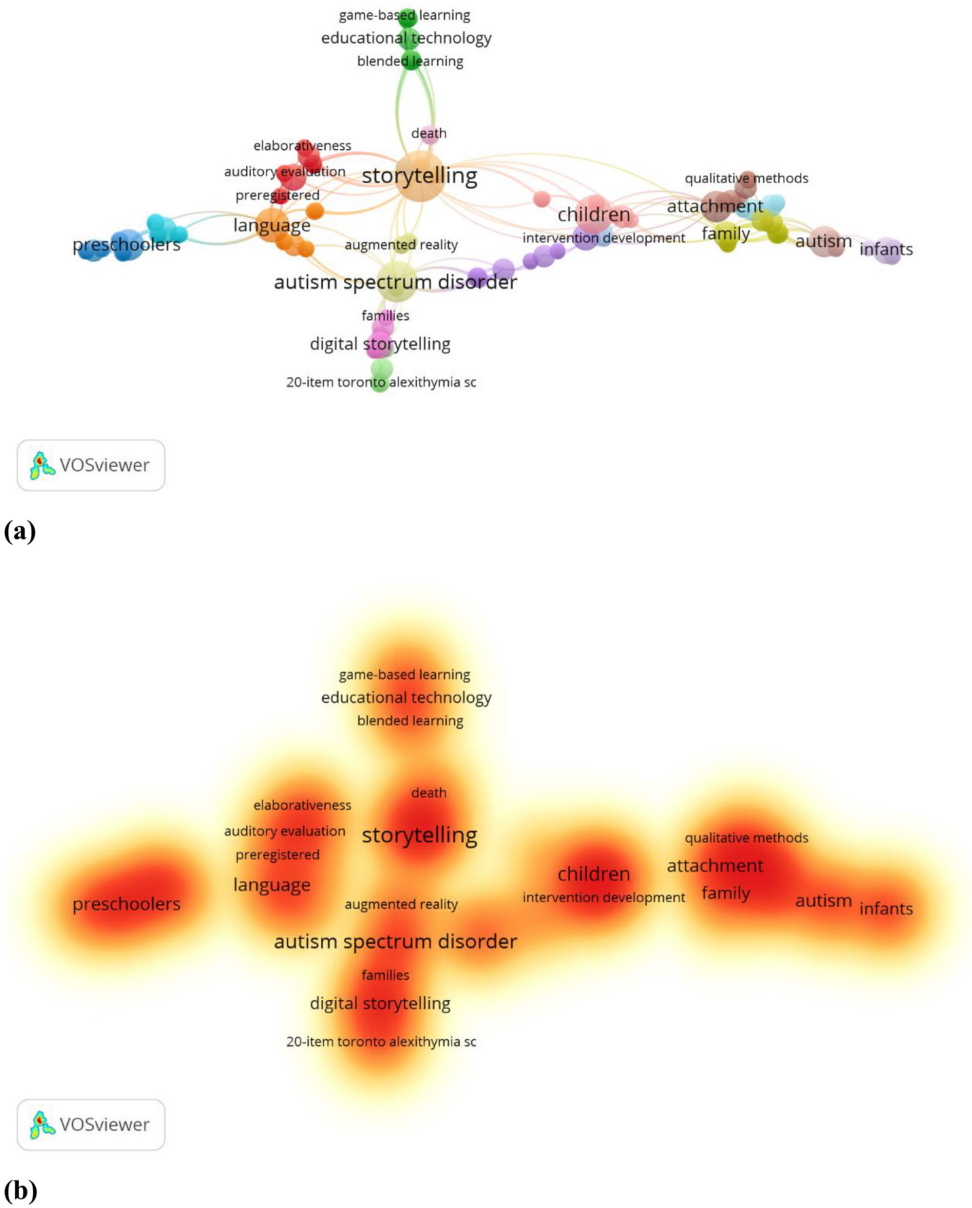
Network visualization of the author's keywords Map; **(a)** network visualization; **(b)** density visualization.

This study focuses on the relationship of parental emotional expression on children's oral narrative ability, and explores related research directions through the keyword co-occurrence analysis map in Figure 5. The map presents a multifaceted and interrelated conceptual network, with lines indicating the co-occurrence of keywords, and the thickness of the lines may indicate the strength of the co-occurrence. In exploring the relationship between “parents' emotional expression” and “children's oral narrative ability,” scholars need to pay attention to a series of closely related potential variables, such as children's emotional state, cognitive ability, family environment, parent-child relationship, etc. (Figure 5). The results of the analysis show that “Storytelling” is a core concept in this field, and is closely related to “Attachment,” “Family” and “Children,” indicating the importance of the family context in the development of children's narrative abilities. Attachment theory suggests that parental emotional support not only affects children's sense of security, but may also enhance their narrative expression through emotional regulation and language input (Bang, 2009; Fitton, 2012; Polovina, 2005). In addition, the strong association between “Language” and “Preschoolers” highlights the importance of oral narrative abilities in early language development, suggesting that positive feedback and emotional value from parents in everyday interactions may promote children's narrative integrity, emotional expression, and story organization. The study also revealed the potential role of “Digital Storytelling” and “Educational Technology,” suggesting that future research could explore how parents can use digital media to enhance children's narrative abilities and optimize their learning experiences with emotional support. The relationship between parental emotional expression in children's narrative development is an emerging topic in this topic, and it is also suggested that future research could explore diverse intervention approaches in conjunction with digital technology to enhance children's narrative expression, especially in culturally diverse and special populations of children, to further validate the mechanism of its influence.

### 3.2 Thematic analysis

Thematic analysis was used to analyze the topics in this study. In the thematic analysis process, we used the six selected articles in **Section 2.3** as our analytical units. These six articles are all published in the Asian region, and focusing on research in the Asian region can explore research goals more deeply and targetedly. First, each study was read in detail to extract specific descriptions and data regarding parental emotional expression and children's oral narrative abilities. Similar descriptions and data were then grouped under the same latent theme. Based on the above narrative theme integration process, this study ultimately extracts the following four core themes: family literacy environment, digital devices, parent-child interaction, and cultural background. The following subsections describe each dimension in detail.

#### 3.2.1 The impact of family literacy environment

In the selected articles, the article “Association between home literacy environment and eye movements during storybook reading: implications for vocabulary and word reading in Chinese preschoolers,” Wen et al. (2024) employed advanced eye-tracking technology combined with quantitative analysis to systematically examine how multiple key dimensions of the family literacy environment (such as language stimulation and interactive reading) influence children's eye movement patterns during storybook reading. This impact was particularly evident in their attention to text regions and their ability to recognize vocabulary and words. The findings revealed that interactive reading, as a core element of the family literacy environment, significantly enhanced children's attention to text and word reading proficiency (β = 0.37, *p* < 0.02). These conclusions resonate with multiple studies on family literacy environments in non-Asian regions, further substantiating the profound influence of the home environment on the development of early reading abilities in children.

Family literacy resources can be divided into traditional literacy resources and electronic product resources. Noguerón-Liu and Driscoll (2021) collected data through interviews, home visits, and children's reading assessments and used a case study approach for their analysis, the results suggest that mothers in bilingual families access literacy resources from schools (e.g., summer programs), community agencies (e.g., libraries), and family networks to improve children's expressive abilities and adaptation to transnational life.

Formal literacy activities refer to structured instructional methods designed to develop children's reading and writing abilities, such as word recognition, phonics, and reading comprehension (Spencer et al., 2013). Formal literacy activities can help children acquire literacy abilities and promote their language development, which indirectly improves progress in narrative expression. Brown et al. (2014) study showed that narrative interventions improved story retelling in at-risk children, which was associated with literacy activities. Spencer et al. (2020a),(b) showed that bilingual instruction enhances preschoolers' oral language abilities, which may include narrative abilities. In addition, a study by Kirby et al. (2021) demonstrated that oral narrative instruction enhanced kindergarten writing, which was closely related to literacy activities.

Informal literacy activities are unstructured daily activities at home, such as reading storybooks together, telling stories, playing word games, or engaging in narrative-related conversations. Informal literacy activities positively influence children's narrative abilities by enhancing language development, comprehension, and interest in reading. Silinskas et al. (2020) found that parents' involvement in family literacy activities, such as storytelling, was effective in improving children's literacy abilities and increasing independent reading abilities resulting in stronger narrative abilities. However, storytelling activities may have different effects depending on the frequency. Bitetti and Hammer (2016) found in a study of Spanish-English bilingual children that mothers' frequent reading of storybooks to their children significantly improved the quality of the children's narratives, and that co-reading activities not only provided linguistic input, but also helped the children to understand the narrative sequence and character development through pictures and storylines. Therefore, the quality of interaction during storytelling was also emphasized as being extremely important in this study as well. Miller-Goldwater et al. (2024) noted that co-reading books can support memory integration in children, especially when the book engages through embedded questions for two individuals, and when both individuals integrate facts while reading. Additionally, in that study it was noted that mothers' storytelling and children's less frequent storytelling (once a month or once a week on average) did not have a significant effect on narrative abilities.

#### 3.2.2 The impact of digital devices

In the selected articles, the article “Digital tools for direct assessment of autism risk during early childhood: a systematic review” by Debarati et al. (2024) systematically reviews various tools and methods for assessing early childhood autism risk using digital technology. The application of digital technology in the field of children's health assessment is becoming increasingly widespread. With the development of new technologies, children's use of digital devices is increasing, which brings new changes to the home learning environment. The intrusion of digital devices, as opposed to traditional literacy activities, has given children flexibility of place, time, and element in the language learning process. In the critical field of children's oral narrative development, numerous studies non-Asian regions have focused on the impact of digital devices.

An analysis by Verhoeven et al. (2020) showed that computer-assisted literacy interventions in preschool and kindergarten environments could improve phonological awareness and reading-related abilities. Additionally, Teepe et al. (2016) showed that technology-enhanced storytelling using digital devices had a positive impact on parent-child interactions and children's productive vocabulary knowledge. A study by Neumann (2016) of 2–4 year olds showed that the frequency of writing with tablets was associated with print awareness, print knowledge, and sound knowledge positively, and non-digital writing activities were positively associated with emergent skills in the home. However, e-reading was not correlated with children's literacy abilities. However, excessive or inappropriate use of digital media can also have negative effects. Passive consumption of digital media (e.g., prolonged television viewing) may be detrimental to narrative development, especially in the absence of interaction. Research has shown that background television noise distracts children and reduces quality interactions with caregivers, which affects language development (Pedrotti et al., 2024). Schwarzer et al. (2022) in the LIFE Child study analyzed 296 children aged 2–5 years and found that more than 1 h of daily screen time was significantly associated with lower percentile rankings in cognitive, language, and socio-emotional skills, with a particularly pronounced decline in language abilities (*b* = −12.88, *p* < 0.01). The impact of digital media activities on children's narrative abilities is not monolithic, but dependent on the mode and context of use. Highly interactive and educational media use may promote narrative abilities, whereas excessive passive consumption may have a negative impact. Parents play a key role in selecting content and engaging in interactions to maximize benefits and minimize risks.

Digital devices, such as tablets, smartphones, and multimedia storytelling apps, have become important mediums in modern homes and educational settings and are widely used in children's language development process. Digital devices often contain rich multimedia content such as video stories, animated storybooks, and interactive language games. These multisensory stimuli not only help children better understand story content and language structures (Takacs et al., 2015), but also provide vivid and interactive learning experiences that help children expand their vocabulary and improve their syntactic understanding, enriching their oral narrative abilities. Meanwhile, many digital storytelling apps help children clarify the beginning, development and ending of a story through structured templates and appropriate prompts, acting as Digital scaffolding (DSC), which effectively improves children's mastery of narrative structure, and thus enhances the coherence and logic of their narratives (Spencer et al., 2020a,b).

The widespread use of digital devices may also have some negative effects on children's narrative abilities. First, overreliance on digital devices may reduce the frequency of direct language interactions between children and adults, undermining the positive effects of shared reading or face-to-face narrative activities on children's language development (Strouse and Ganea, 2017). Second, while structured narrative templates in digital devices can help children construct stories, their long-term use may limit children's ability to create and express themselves freely, making them more inclined to rely on established templates and lacking spontaneous creative narratives (Kucirkova et al., 2015). In addition, rich interactive elements (e.g., animation and sound effects) in digital devices may distract children's attention from deeper understanding of narrative content and linguistic details by focusing more on the surface features of the story (Bus et al., 2015), which reduces the quality of narrative expression.

#### 3.2.3 The impact of parent-child interaction

In the selected articles, Hsieh (2023) in the article “This is My Favorite Book': Exploring Children's Perspectives on Books and Reading” utilized both qualitative and quantitative research to reveal children's desire for shared reading with adults and their wish for greater participation and choice. This highlights the crucial role of parent-child interaction in reading and reminds parents and educators to foster children's reading initiative. Another study, Hong et al. (2022) in “Meta-Analysis of RCTs of Technology-Assisted Parent-Mediated Interventions for Children with ASD” evaluated the effectiveness of technology-assisted parent-mediated interventions for children with autism. It found these interventions had some impact on emotion recognition but showed limited effectiveness in social communication and language development, suggesting interventions need to be more targeted and personalized. Also, numerous studies non-Asian regions have further confirmed the profound impact of parent-child interaction patterns and targeted interventions on children's reading and social-language development.

Children's cognitive and language development relies on the process of interaction with adults or more experienced peers. High-quality parent-child language interactions can provide children with rich and varied language input and promote the development of vocabulary building and language comprehension (Hart and Risley, 1995). Rowe (2012) study showed that interactions in which parents use a more varied vocabulary with appropriate language extensions can lead to richer narratives and more precise vocabulary use by children. In addition, positive emotional support and encouragement not only create a favorable emotional climate for children, but also significantly increase their initiative and self-confidence in participating in oral narrative activities, thus effectively enhancing narrative motivation and narrative quality (Fivush and Nelson, 2006). In the specific narrative process, parents can build up “narrative scaffolding” through open-ended questions, detailed descriptions, etc., to help children gradually grasp the structure of the story (beginning, development, climax, and end), which effectively enhances their narrative logic and coherence (Haden et al., 1997).

However, the low quality of parent-child interactions (e.g., single language input, lack of or oversimplified responses) may result in children's expressive language abilities being limited by a monotonous vocabulary, poor content, and a lack of logic in their narratives (Hoff, 2006). In addition, low quality emotional interactions (e.g., negative parental feedback or neglectful behaviors) may result in low narrative motivation or even narrative anxiety, leading to avoidance of or passive participation in oral narrative activities and weakening the development of narrative ability (Laible, 2011). At the same time, if parents are too dominant in their interactions (e.g., frequent instructions or correction of errors), children may have difficulty organizing stories on their own, limiting their narrative creativity and spontaneity (Tamis-LeMonda et al., 2001).

#### 3.2.4 The impact of cultural background

In addition to the hot topics of research mentioned above, there are significant differences in the oral narrative styles of children from different cultural backgrounds. Among the selected articles, Sebastian et al. (2019) in “Parents, Television, and Children's Emotional Expressions: A Cross-Cultural Multilevel Model” conducted cross-cultural comparisons to examine the effects of parenting styles and television media on children's emotional expression, highlighting cultural differences and underscoring the role of cultural context in children's emotional socialization. This study suggests that research interventions should account for cultural factors. Anita et al. (2011) in “Development of the language subtest in a developmental assessment scale to identify Chinese preschool children with special needs” focused on developing language assessment tools for children with special needs within the Chinese cultural context. Through rigorous procedures, they ensured the tool's cultural adaptability and measurement validity, providing a reference for assessment tool development in China and across cultures. Furthermore, research non-Asian regions has also examined the relationship between cultural background and children's oral narrative abilities.

Children in European and American cultural settings tend to have an individualistic orientation, with more emphasis on personal emotions and individual experiences in their narratives. Children from East Asian cultures, on the other hand, showed a stronger collectivist orientation, focusing more on interpersonal interactions and social norms in their narrative content (Minami, 2008). This difference further influences children's choices of story structure, character relationships, and plot expression. In addition, parental interaction styles also show differences depending on cultural background and significantly affect children's narrative expression. For example, North American and European families are more likely to adopt an elaborative style, which provides rich emotional experiences and detailed descriptions in their interactions, thus effectively enhancing children's narrative structure and language complexity (Fivush and Wang, 2005). In contrast, Asian families have a more conservative style of interaction, with parents focusing more on emphasizing social values and tending to adopt a concise narrative language style (Wang and Leichtman, 2000).

## 4 Discussion

This study combines bibliometric and thematic analyses to systematically explore the influence of parental emotional expression on children's oral narrative ability, and reveals the future development trend in this field based on existing research. The findings not only verified the key role of parental emotional expression in children's language development, but also revealed the research hotspots, international cooperation patterns and interdisciplinary integration trends in this field.

Bibliometric analyses show that the field is moving toward interdisciplinary convergence, and the combination of artificial intelligence, psychology, and neuroscience is driving more accurate assessment of children's oral narrative ability and intervention strategies (Tables 4–6). Publication Trends show that the field experienced high-impact bursts in 2015 and 2021, but the declining citation rates in recent years may reflect changes in research hotspots, uneven research quality, or fluctuations in the field's attention (Table 7, Figure 3). In terms of academic output, the internationalization of this research topic has gradually increased, with European and American scholars still dominating, but research contributions from Asia and emerging countries are increasing, and the trend of global cooperation is becoming more and more significant (Tables 8, 9). It is noteworthy that the field is still mainly dominated by Europe and the United States, but there has been a tendency to expand to developing countries (Table 10, Figure 4). In addition, the present study demonstrated that parental emotional expression, parent-child interaction, cultural background, and technological interventions collectively influence children's oral narrative ability, but existing studies have paid less attention to the interaction of these factors (Figure 5).

Thematic analysis further validated the relationship between parental emotional expression on children's narrative development. Parental emotional expression not only directly affects children's oral narrative ability, but also displays a multilevel mechanism of action moderated by the family literacy environment, the use of digital devices, parent-child interactions, and the cultural background. The family literacy environment provides children with initial language stimulation and narrative experiences, digital devices enrich children's language input and output channels with emerging media, the quality of parent-child interactions directly affects children's emotional experiences and narrative motivation, and the cultural background shapes children's narrative styles and thematic preferences. Together, these factors determine the breadth and depth of the influence of parental emotional expression on children's oral narrative ability.

Numerous studies have shown that a rich family literacy environment positively contributes to children's language development and narrative ability. Specifically, the number of accessible reading materials in the home, the frequency of shared reading activities, and positive parental guidance of text in daily life significantly enhance children's vocabulary and syntactic complexity (Dahl Leonard et al., 2025; Galea, 2024). On top of this, positive emotional expressions, such as enthusiasm and empathy, displayed by parents during the reading process further stimulated children's interest and willingness to participate in narrative activities. However, poor literacy resources and a negative emotional climate may inhibit children's self-expression during the narrative process, limiting the development of their narrative ability.

At the same time, the widespread use of digital media has brought about complex and contradictory influences on the construction of children's narrative ability. On the one hand, digital storytelling apps with high interactivity and rich content can facilitate children's comprehension and retelling of storylines through multichannel sensory stimulation (Choi et al., 2025; Ye et al., 2024). In particular, children's narrative motivation and confidence in expression may be significantly enhanced when parents accompany children with positive emotions in the use of digital devices. On the other hand, over-reliance on or inappropriate use of digital devices may diminish direct oral communication and emotional interactions between children and their parents, thereby reducing high-quality oral input and ultimately having an inhibitory effect on narrative ability (Sundqvist et al., 2024; Mills, 2020). These results suggest that the way digital technology is used and its mode of integration with parent-child interactions in family contexts has a key moderating role in the development of children's narrative ability.

In addition, parent-child interactions also play a non-negligible role in the formation of children's narrative ability. High-quality interactions, such as empathic listening, extended questioning, and positive feedback, not only facilitate children's use of richer linguistic resources in their narratives, but also contribute to the construction of more coherent and logical story structures (Rowe, 2022; Gardner-Neblett and Iruka, 2024). Positive emotional expression further enhances complexity in children's linguistic output and increases their sense of self-efficacy in narrative activities. In contrast, the absence of emotional support or excessive parental dominance in narratives may lead to a decline in children's narrative motivation, which in turn leads to homogenization of narrative content and a lack of emotional depth (Mills, 2020; Gonzalez, 2025). Thus, parent-child interactions not only affect the quantitative level of narrative ability, but also have a profound impact on the level of narrative quality and emotional expression.

Further, the cultural background, as an important part of the macro-socialization mechanism, also plays a profound role in shaping children's narrative styles. In European and American cultures, parents often encourage children to narrate their personal emotional experiences in a self-centered manner, an orientation that enriches narrative content and enhances the depth of emotional expression (Gardner-Neblett and Iruka, 2024). In East Asian cultures, parents emphasize social responsibility and collective identity, and children's narratives tend to focus on social relationships and norm-following (Gonzalez, 2025). Cultural background not only shapes the form and frequency of parental emotional expression, but also indirectly influences the developmental path of children's narrative ability through the socialization process. This cultural difference suggests that cross-cultural variability and its underlying mechanisms need to be fully considered when understanding the influence of family factors on children's narrative ability.

In summary, the family literacy environment, digital devices, parent-child interactions, and cultural background play multilayered and intertwined roles in the development of children's narrative ability. Although existing studies have provided rich empirical evidence, there is still a lack of theoretical integration, limited exploration of mechanisms, and cross-cultural comparisons in this area. In order to further deepen the understanding of the developmental mechanisms of children's narrative ability and to enhance the application value of the research, future studies can explore the following directions. First, interdisciplinary cooperation should be strengthened to promote the integration and application of neuroscience and psychology in order to optimize the assessment tools and intervention methods for children's language development. Second, it is necessary to improve the rigor of research methods, enhance the reproducibility of data, and summarize and integrate existing research results through systematic reviews, so as to provide a more solid foundation for theoretical construction. Third, promoting international cooperation is also an important direction for the future, especially in expanding the coverage of research on developing countries and culturally diverse populations, so as to enrich the global perspectives of the field and enhance the generalizability of the research results. In addition, combining innovative interventions with digital technology and exploring intelligent and personalized programs to promote children's language development are expected to open up new paths of practice. Finally, research on the integrated mechanism of emotional expression, parent-child interaction, and cultural factors needs to be further deepened to optimize family education strategies to more effectively support the development of children's oral narrative abilities. Through the above multi-dimensional expansion, future research is expected to make more far-reaching progress in theory deepening and practical application, contributing to the construction of a scientific guidance system for global children's language development.

## 5 Recommendations

Based on the bibliometric analysis, this study explores the research trends, internationalization, interdisciplinary integration, and future directions of the field. However, there are still some limitations, such as limited sample coverage, small sample size, and a single research method. Future research should expand the sample coverage, adopt mixed research methods, use dynamic analysis techniques and increase practical application studies to improve the scientific, systematic and practical impact of the research, so as to promote the development of the field toward a more precise, globalized and practice-oriented development.

## 6 Conclusion

This study screened the literature through PRISMA principles, and then explored the research trends, academic influence, international cooperation patterns, dynamic evolution of interdisciplinary development, and analysis of research hot topics in the field of the relationship between parental emotional expression and children's oral narrative ability based on bibliometric analysis and thematic analysis. The results indicated that the field experienced research peaks in 2015 and 2021, reflecting the stage-by-stage academic interest in the topic. However, the decline in citations in recent years may indicate the change of research hotspots and the adjustment of research methods, and the theoretical framework and research design need to be further optimized in the future to maintain the academic influence of the field. Further, the research on this topic is developing toward interdisciplinary integration, with the combination of psychology, neuroscience, artificial intelligence technology and other fields providing new perspectives on the assessment and intervention of children's oral narrative ability. Meanwhile, there is a growing trend of international cooperation. Although European and American scholars still dominate, research contributions from Asia, Latin America, and other developing countries have gradually risen, indicating that the field is evolving toward a globalized and diversified research pattern. In addition, this study suggests that parental emotional expression, parent-child interaction, cultural background and technological intervention jointly affect children's language development, but the interaction of these factors still needs to be further explored in order to optimize family education and intervention strategies.

Parents' emotional expression patterns directly affect children's oral narrative abilities; positive emotional expression promotes language richness, whereas negative emotional expression may inhibit language development. High-quality parent-child interactions effectively moderated the effects of parental emotional expression on children's narrative ability, while cultural context and technological interventions played an indirect role. Future research should continue to focus on children's language development from a cross-cultural perspective and explore the application of cutting-edge technologies, such as artificial intelligence and natural language processing, in children's language assessment and intervention, in order to optimize family education strategies and promote the continued development of the field.

Overall, this study reveals the current state of research in this field and the thematic categorization of this research topic, and provides a reference for future academic exploration. Future research should further expand in the areas of theoretical deepening, methodological innovation, cross-cultural research, and digital interventions, in order to promote the continued development of the field and provide more accurate and scientific guidance for children's language development.

## Data Availability

The original contributions presented in the study are included in the article/supplementary material, further inquiries can be directed to the corresponding author.
